# Key separable events in the remodelling of the pharyngeal arches

**DOI:** 10.1111/joa.13850

**Published:** 2023-02-23

**Authors:** Subathra Poopalasundaram, Jo Richardson, Anthony Graham

**Affiliations:** ^1^ Centre for Developmental Neurobiology, King's College London London UK; ^2^ School of Life Sciences University of Sussex Brighton UK

**Keywords:** hyoid arch, pharyngeal arch, pharyngeal morphogenesis, pharyngeal pouch

## Abstract

The pharyngeal arches are a series of bulges on the lateral surface of the embryonic head. They are a defining feature of the most conserved, the phylotypic, stage of vertebrate development. In many vertebrate clades, the segmental arrangement of the pharyngeal arches is translated into the iterative anatomy of the gill arches. However, in amniotes the pharyngeal arches undergo a rearrangement during development and the segmental organisation of the pharynx is lost. This remodelling involves the expansion of the second arch which comes to overlie the more posterior arches. A transient sinus forms between the expanded second arch and the posterior arches, that is then lost, and the posterior arches are internalised. The morphogenesis of the second arch has been viewed as being central to this remodelling. Yet little is known about this process. Therefore, in this study, we have characterised the development of the second arch. We show that as the second arch expands, its posterior margin forms a leading edge and that the mesenchymal cells subjacent to this are in an elevated proliferative state. We further show that the posterior marginal epithelium is the site of expression of three key developmental signalling molecules: *BMP7*, *FGF8* and *SHH*, and that their expression continues throughout the period of expansion. Using a novel approach, we have been able to simultaneously inhibit these three pathways, and we find that when this is done the second arch fails to establish its caudal projection and that there is a loss of proliferation in the posterior mesenchymal cells of the second arch. We have further used this manipulation to ask if the internalisation of the posterior arches is dependent upon the expansion of the second arch. We find that it is not—the posterior arches are still internalised when the expansion of the second arch is curtailed. We further show that while the collapse of the sinus is dependent upon thyroid hormone signalling, that this is not the case for the internalisation of the posterior pouches. Thus, the internalisation of the posterior arches is not dependent on the expansion of the second arch or on the collapse of the sinus. Finally, we show that the termination of expansion of the second arch correlates with a burst of morphogenetic cell death suggesting a mechanism for ending this. Thus, while it has long been thought that it is the morphogenesis of the second arch that drives the remodelling of the pharyngeal arches, we show that this is not the case. Rather the remodelling of the pharyngeal arches is a composite process that can split into contemporaneous but separate events: the expansion of the second arch, the internalisation of the posterior arches and the collapse of the sinus.

## Graphical abstract

BMP7 (A), FGF8 (B) and SHH (C) are expressed at the posterior margin of the second arch during its period of expansion. Expression of these genes can also be seen in other regions of the embryo including the developing limb buds. Sections through the expanding second arch at stage 24 show the localised epithelial expression of each of these ligands.

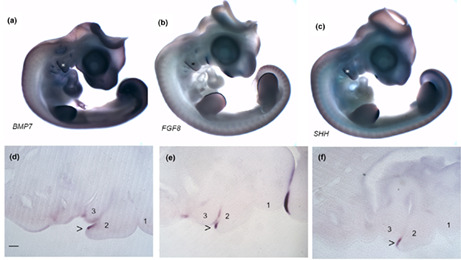

## 1 INTRODUCTION

The pharyngeal arches are a significant feature of vertebrate embryos (Graham & Richardson, 2012). They present as a series of bulges formed on the lateral surface of the head. The first, and key, event in the establishment of the pharyngeal arches is the formation of outpocketings within the endoderm, the pharyngeal pouches. The first two form at the same time with the others forming later and sequentially (Crump et al., 2004; Shone & Graham, 2014). The pouches contact the overlying ectoderm, which invaginates to meet them forming the pharyngeal clefts and it is this apposition between the pouches and clefts that will define the margins of each pharyngeal arch. These preformed units are then populated by neural crest cells and mesoderm. These different embryonic groups will differentiate to form a range of tissues: the neural crest gives rise to skeletal and connective tissues, the mesoderm to muscle and endothelium, the ectoderm to the skin and sensory neurons and the endoderm to the lining of the pharynx and specialised organs (Graham & Richardson, 2012).

In many vertebrate clades, this embryonic organisation is translated into the later segmental organisation of the gill‐bearing branchial arches (Kardong, 2012; Romer, 1971). In amniotes, however, the pharyngeal arches undergo a complex remodelling and there is no simple correspondence between the embryonic segments and the later anatomy. In this group, once the full complement of arches; of which there are five, has been realised, this region undergoes a complex remodelling that results in the expansion of the second pharyngeal arch and loss of the posterior segments (Poopalasundaram et al., 2019; Richardson et al., 2012). This has been appreciated for a long time, but it has been little studied.

A standard description of pharyngeal remodelling would be as follows. At a given time in development (stage 20 in the chick, E10 in mouse, Cs15 in humans) the second pharyngeal arch expands caudally covering the more posterior arches (Graham et al., 2019; Kaufman, 1992; Poopalasundaram et al., 2019; Schoenwolf, 2014). The leading edge of the expanding second arch then fuses with the underlying tissue and thus the more posterior arches are internalised. Initially, there exists a cavity, the cervical sinus, between the inner surface of the expanded second arch and the outer surfaces of the posterior arches, but this subsequently collapses. Thus, with time, the segmental organisation of the posterior pharyngeal region is lost. The emphasis here is that it is the expansion of the second arch that drives this remodelling, and the other events, the internalisation of pouches 3 and 4 and the collapse of the sinus, are dependent upon this.

The aim of this present study is to provide an understanding of the expansion of the second arch and how that relates to the remodelling of the posterior arches. In particular, the second arch undergoes a protracted period of expansion, and we have little knowledge of how this is directed and maintained. We have also not been able to experimentally manipulate the expansion of the second arch to test if the internalisation of the posterior arches is dependent upon this event. Finally, we have no comprehension of why the expansion of the second arch terminates.

## 2 METHODS

### 2.1 Chick embryos

Fertilised hen's eggs were incubated at 38°C and staged according to Hamburger and Hamilton (Hamburger & Hamilton, 1951).

### 2.2 Sub‐blastodermal injection of chick embryos

Hen's eggs were incubated to either stage 18 or stage 22. Eggs were sprayed with 70% (v/v) ethanol and allowed to dry. A small volume (2/5 mL) of albumin was removed from the egg to allow embryos to sink. Eggs were windowed and 50 μL volumes of either drug or vehicle controls were injected under the embryo within the yolk. Amiodarone was used at a final concentration of 10 μM, methimazole at 20 mM, dorsomorphin at 10 μM, SANT‐1 at 10 μM and SU5402 at 13.6 μM. Sub‐blastodermal injection allows the drug concentration to be maintained and lower doses of the drugs can also be used as the drug is contained within the yolk. Five hundred microlitre of penicillin/streptomycin diluted in phosphate buffered saline, pH 7.4 (PBS) was added and the eggs sealed with Sellotape and returned to the incubator until the relevant developmental stage was attained. Embryos were dissected in PBS and fixed in 4% (v/v) formaldehyde (FA) in PBS.

### 2.3 Whole mount in situ hybridisation

The amniotic membranes were removed, and the embryos were fixed in 4% (v/v) FA in PBS overnight. Embryos were washed twice in PBST (PBS containing 0.1% (v/v) Tween‐20), then dehydrated through 25%–100% methanol in PBST. Embryos were either stored in 100% methanol at −20°C or processed for in situ hybridisation. Embryos were then rehydrated in 75%–25% methanol in PBST and treated with 10 μg/mL Proteinase K in PBS for 20 min, postfixed with 4% (v/v) FA and 0.2% (v/v) glutaraldehyde for 20 min in PBST, and then washed twice in PBST. The embryos were incubated at 70°C in hybridisation buffer (50% [v/v] formamide, 5× SSC pH 4.5, 2% [w/v] SDS, 2% [w/v] Blocking reagent [Roche]) for 1 h, followed by overnight at 70°C in digoxigenin‐labelled riboprobes, diluted 10 μL in 1 mL hybridisation buffer (*BMP7*; Houston et al., 1994), *FGF8* (Crossley et al., 1996) *SHH* (Roelink et al., 1994). Post‐hybridisation washes were performed twice in solution X (50% [v/v] formamide, 2× SSC, pH 4.5 and 1% [w/v] SDS) rapidly and then three times for 30 min at 70°C. Embryos were then washed twice for 10 min in MABT (100 mM maleic acid, 150 mM NaCl, 1% [v/v] Tween‐20; pH 7.5) and blocked with blocking solution (2% [w/v] BBR [Roche]/20% [v/v] goat serum/MABT) for 1 h at room temperature and incubated overnight at room temperature in anti‐DIG‐AP antibody (Roche) diluted 1:2000 in blocking solution. Embryos were washed thoroughly with MABT and equilibrated in NTMT (100 mM NaCl, 100 mM Tris–HCl, pH 9.5, 50 mM MgCl_2_ and 0.1% [v/v] Tween‐20). Alkaline phosphatase activity was detected using NBT and BCIP in NTMT (10 μl per ml NTMT), initially at 37°C then at room temperature. The reaction was stopped by washing in MABT and fixing in 4% (v/v) FA in PBS. Gene expression was either examined as whole mounts or embryos were embedded in 20% (w/v) gelatin in PBS and sectioned at 50 μm using a vibratome.

### 2.4 Whole mount immunostaining

Immunostaining was done using standard whole mount immunostaining protocol. Fixed embryos were washed three times 30 min in PBSTx (PBS containing 1% [v/v] TritonX‐100) and blocked with 10% (v/v) goat serum/PBSTx/2% (w/v) NaN3 for 90 min, followed by an overnight incubation at room temperature in primary antibody, rabbit anti‐phospho‐SMAD1/5/8 at 1:300 (Cell Signalling #13820). Embryos were washed three times in block and then incubated overnight at room temperature in an appropriate Alexa conjugated secondary antibody (Molecular Probes; 1:1000). Embryos were then washed in PBSTx, bisected or sectioned and photographed using a fluorescence microscope.

### 2.5 LysoTracker staining

Embryos were dissected in PBS and transferred to individual wells in a 12‐well plate that contained 2.0 mL PBS per well. 2.5 mL PBS was warmed to 37°C and 25 μL LysoTracker red (molecular probes L‐7528 RED) added. 1.75 mL PBS was removed from each well and 250 μL LyosTracker red in PBS was added to each well. The plate was covered with foil and incubated at 37°C for 30 min. The embryos were rinsed four to five times gently with PBS, fixed in 4% (v/v) FA in PBS overnight at 4°C, rinsed in PBS, dehydrated in 100% methanol to remove any background staining, and viewed under a fluorescence microscope to obtain whole mount images. Stained embryos were also embedded in gelatin, sectioned and analysed by microscopy.

## 3 RESULTS

### 3.1 The expansion of the second arch

**FIGURE 1:**
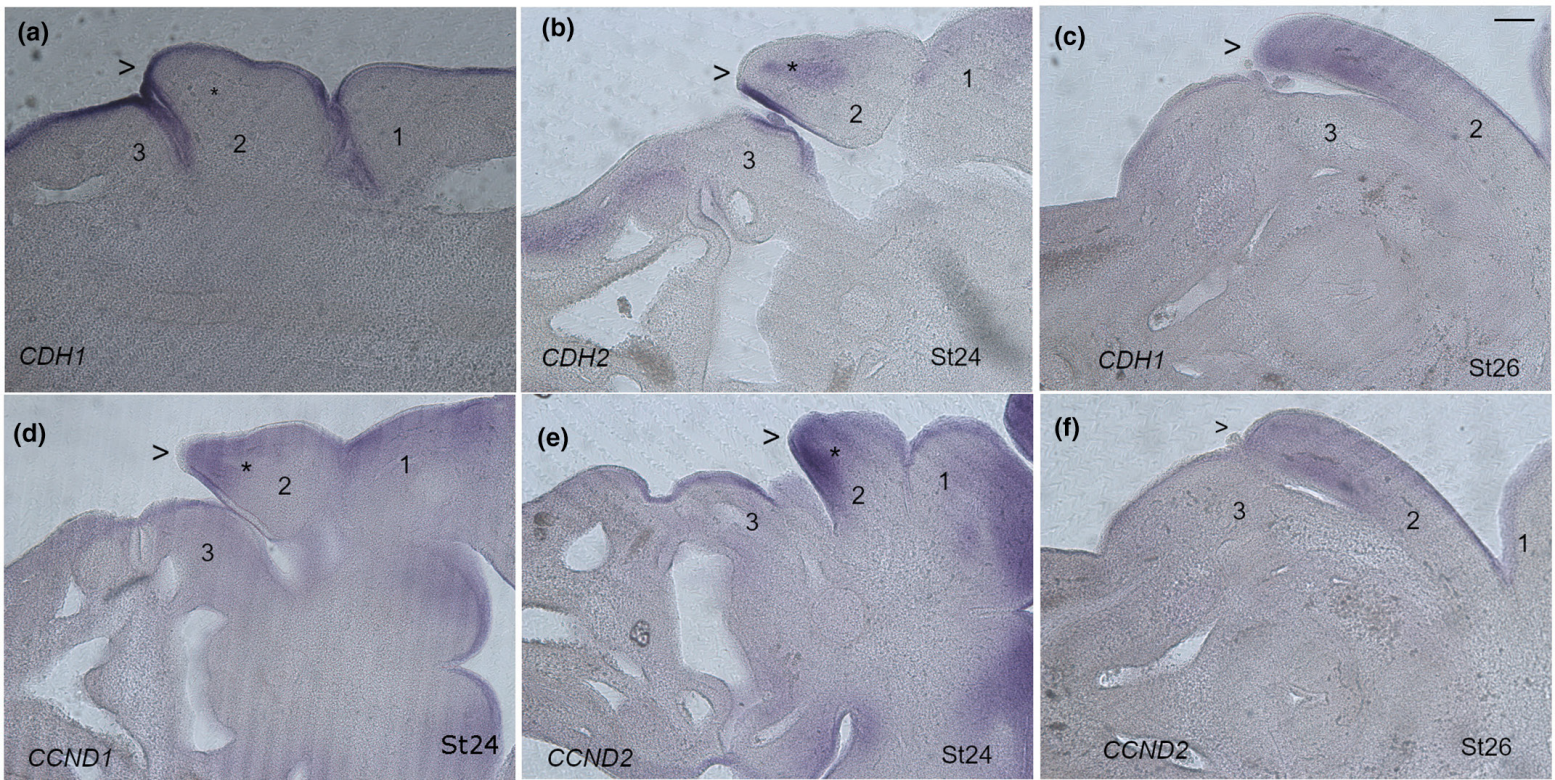
Overview of the expansion of the second arch. *CDH1* (A, C) and *CDH2* (B) expression highlights the morphogenesis of the epithelium of the second arch (2). At stage 24 (a, b) the posterior margin (<) can be seen to be bulging caudally. At this stage, *CDH2* expression also highlights the position of the mesodermal core (*). By stage 26 (c) the caudal projection of the second arch is more developed and can be seen to extend over the posterior pharynx. *CCND1* and *CCND2* (d, e) expression highlights regions of elevated proliferation. At stage 24, there is pronounced expression of *CCND1* and *CCND2* in the mesenchyme underlying the poster margin (<) of the second arch. However, there is less expression in the mesodermal core (*). Elevated regional expression of *CCND2* in the mesenchyme underlying the posterior margin of the second arch is still seen at stage 26 (f). In all panels, anterior is to the right. The pharyngeal arches are numbered, 1, 2 and 3. < − indicates the leading edge of the second arch. *The position of the mesodermal core. Scale bar—100 μm.

Initially the pharyngeal arches are alike in proportion. However, as embryogenesis proceeds, the development of the second, or hyoid, arch (PA2) becomes markedly different from that of the others. From stage 19 onwards, in chick, it expands, as its posterior margin, which is formed from the second pharyngeal pouch and cleft, breaks away and projects caudally (Poopalasundaram et al., 2019; Shone & Graham, 2014). The general morphology of the second arch during the period of expansion has been little analysed and therefore we used *CDH1* (*E‐cadherin*) and *CDH2* (*N‐cadherin*) expression to highlight the epithelium. We find that during the phase of expansion, the second arch adopts a polarised morphology. Thus, by stage 24 the second arch has a pronounced leading edge at its posterior margin (Figure 1a,b). This gets exacerbated with time, and by stage 26 the second arch is projecting caudally over the more posterior region of the pharynx (Figure 1c). This continues until stage 28 when the leading edge of the second arch is no longer morphologically evident. We also mapped patterns of proliferation in the second arch during the expansion using Cyclin D expression (Towers et al., 2008). *CCND1* (*cyclin D1*) and *CCND2* (*cyclin D2*) can be seen to be expressed at high levels in the mesenchyme underlying the posterior margin of the second arch at stage 24 (Figure 1d,e). The pattern of staining also indicates that this is primarily associated with the neural crest component of the arches as there is less staining in the mesodermal core (Figure 1d,e). The mesodermal core is highlighted by the expression of *CDH2* (Figure 1b). Higher levels of *CCND2* are maintained in the mesenchyme immediately subjacent to the epithelium of the leading edge at stage 26 (Figure 1d). Thus, localised high levels of proliferation are found in the posterior mesenchyme during the period of expansion of the second arch.

### 3.2 The epithelial signals driving the expansion of the second arch

**FIGURE 2:**
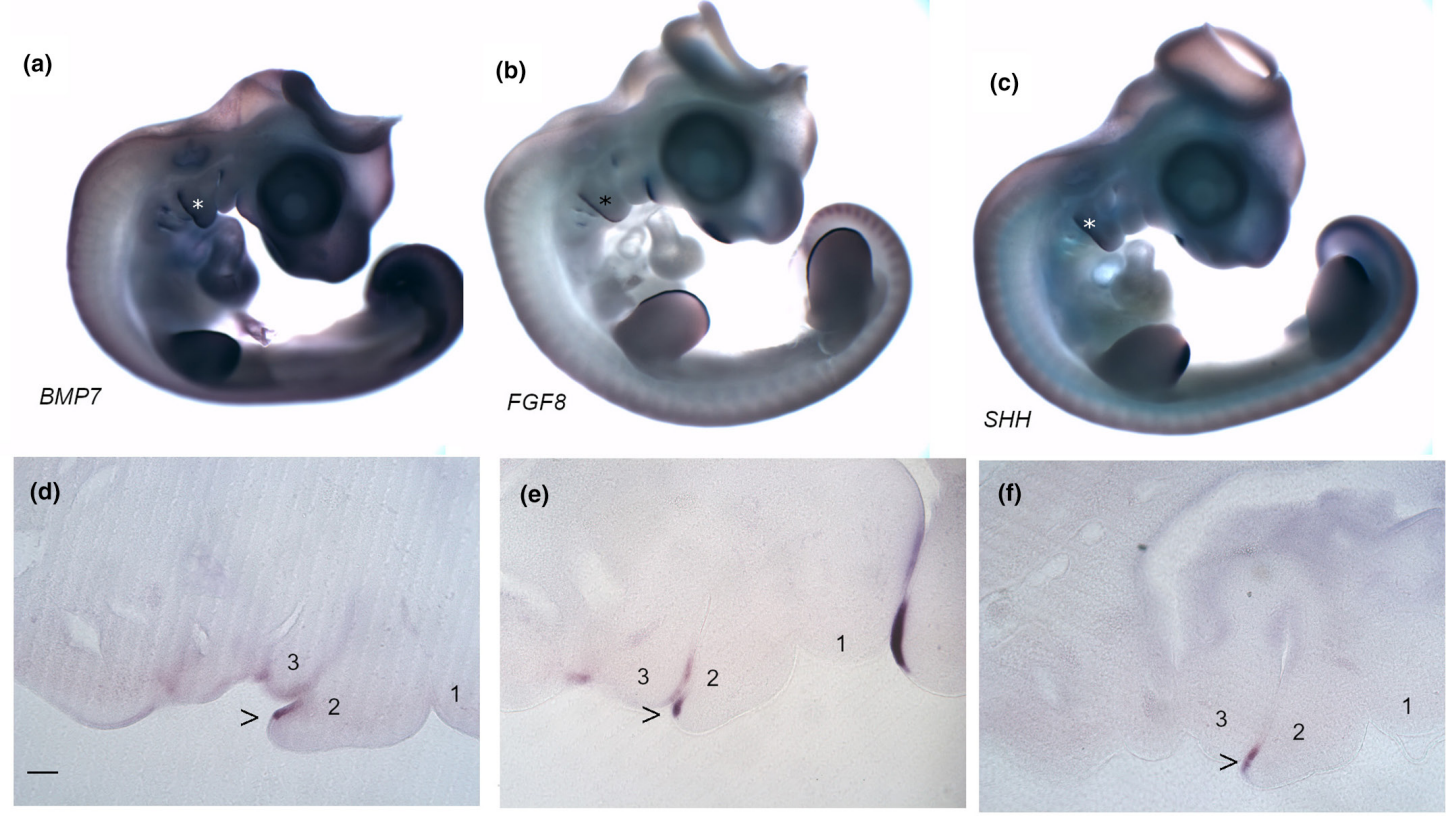
*BMP7*, *FGF8* and *SHH* are expressed at the posterior margin of the second arch during the period of expansion. Whole mount in situ hybridisation at stage 24 showing the expression of *BMP7* (a), *FGF8* (b) and *SHH* (c). Expression can be seen at the posterior margin of the second arch which is indicated by an (*). Expression of these genes can also be seen in other regions of the embryo including the developing limb buds. Sections through the expanding second arch at stage 24 show the localised expression (>) of each of these ligands—*BMP7* (d), *FGF8* (e) and *SHH* (f)—in the epithelium that lies inward, the endoderm, from the leading edge of the second arch. In all panels, anterior is to the right. The pharyngeal arches are numbered, 1, 2 and 3. Scale bar—100 μm.

The posterior margin of the second arch has been shown to express key signalling molecules: *BMP7*, *FGF8* and *SHH* prior to the expansion of the second arch, in both chick and mouse (Gavalas et al., 2001; Veitch et al., 1999; Wall & Hogan, 1995). We have therefore further characterised the expression of these genes during the period of the expansion of the second arch. We find that all three signalling molecules continue to be expressed at high levels at the posterior margin and that this is maintained. This is shown at stage 24 (Figure 2a–c). The sections demonstrate that these genes are all expressed in a similar domain within the epithelium, just internal to the leading edge (Figure 2d–f).

These three signalling molecules are all known to be able to drive proliferation and we therefore sought to inhibit these pathways to determine if that could halt the expansion of the second arch. Inhibiting three pathways at the same time during development is a difficult task. However, we have developed a novel approach in chick that allows for this manipulation. Our protocol involves the sub‐blastodermal injection of small molecule pharmacological modulators of signalling pathways into the yolk compartment of chick eggs. Such molecules have been used in the chick before, but this has usually been done with beads, in which case it is often very difficult to control the concentration seen by responding cells, or in new cultures of very young embryos, which restricts the analysis to a very small developmental window. The advance here is that we can accurately control the dose of the drug, and this can be done at a broad range of developmental stages. The yolk compartment is a defined and relatively small space (~10 mL) and thus one does not need to use large quantities of these compounds to reach the concentrations at which they are effective. These molecules are then efficiently taken up by the embryo, which is developing on top of that yolky mass, allowing us to accurately modulate signalling pathways, and to precisely control the timing of these interventions *in ovo*.

To establish that this approach works, we blocked each of these pathways with specific antagonists and assessed the consequence on known outcomes for each. The control for each of these interventions was injection with the carrier for the drug, DMSO. To block BMP signalling, we used injections of 10 μM dorsomorphin (Yu et al., 2008) and there is a clear loss of pSMAD staining in the treated versus the control embryos (Figure S1). FGF signalling is important for many events in the embryo including the formation of somites and the expansion of the mesencephalon. We found that in embryos treated with 17 μM SU5402 (Mohammadi et al., 1997), there were expected morphological alterations (Figure S1). The midbrain did not expand, and the formation of the somites was severely perturbed, resulting in a shortened axis. Treatment of embryos at this dosage of SU5402 also resulted in loss of expression of the FGF downstream gene, SPRY2 (data not shown). To block SHH signalling we used the smoothened antagonist SANT1 (Frank‐Kamenetsky et al., 2002). In embryos injected with 10 μM SANT1 there was a clear loss of *PTCH1* staining from both the pharyngeal arches and the limbs (Figure S1).

**FIGURE 3:**
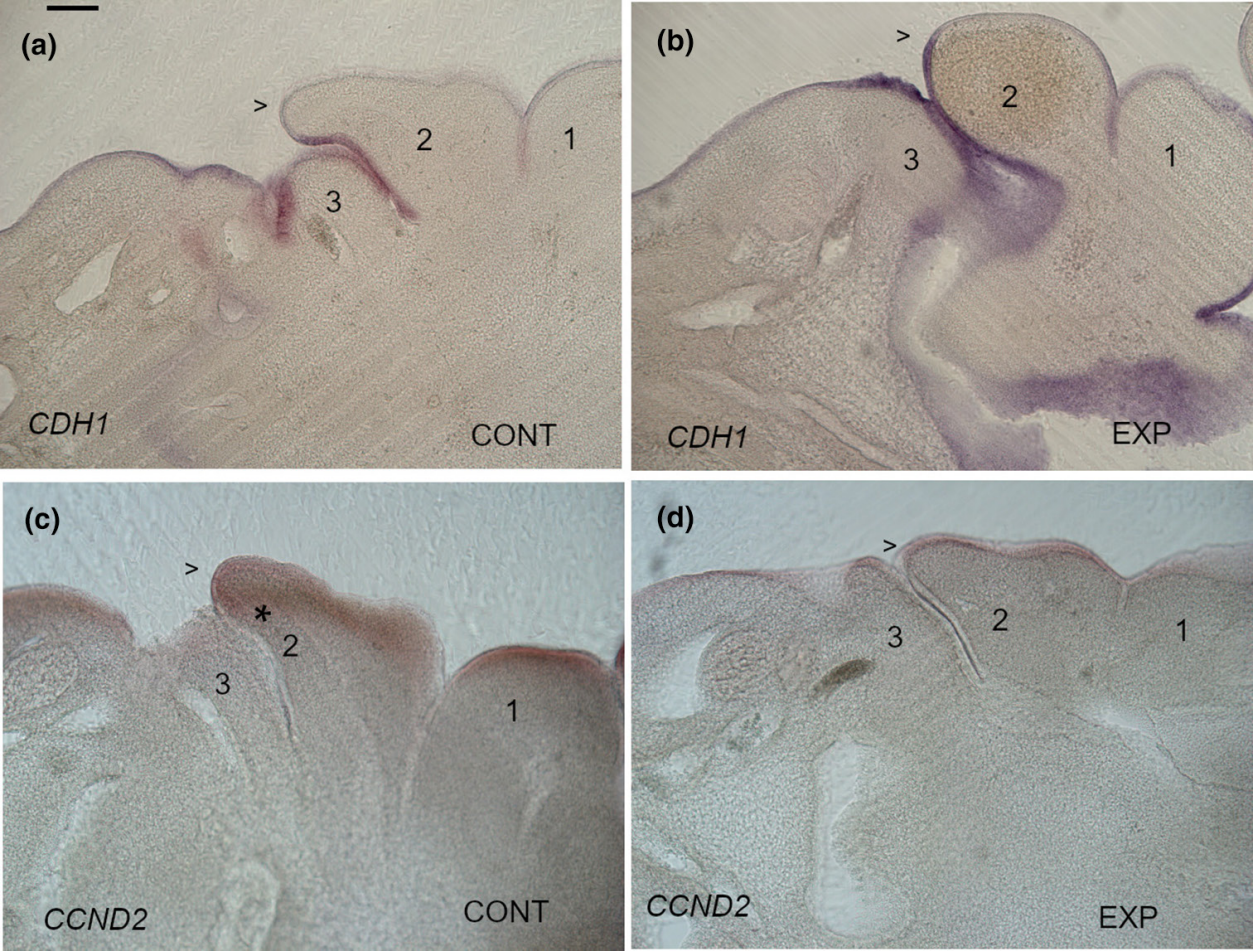
Triple blocking BMP, FGF and SHH signalling results in a curtailment in the expansion of the second arch and loss of proliferation in the arch mesenchyme. In embryos that have been injected with DMSO (a) the second arch can be seen to establish its normal posterior projection (>) as revealed via *CDH1* expression. However, in embryos that have receive the triple block cocktail (EXP) (b), the second arch has failed to project caudally and has an altered more rounded morphology (>). In embryos that received a DMSO injection (c), elevated proliferation as evidence through *CCND2* expression (*) is seen in the mesenchyme close to the epithelial margin. However, this is lost from embryos (EXP) (d) that received the triple block. In all panels, anterior is to the right. The pharyngeal arches are numbered, 1, 2 and 3. Scale bar—100 μm.

A further advantage of this protocol is that it allows one to target multiple signalling pathways simultaneously. We therefore made a cocktail of these small molecule antagonists and injected blockers of all three pathways simultaneously at stage 22, which is relatively early during the period of expansion. Dorsomorphin and SANT1 were both used at 10 μM while SU5402 was used at the slightly reduced concentration of 13 μM to facilitate the making of the cocktail. However, we did confirm that similar morphological changes as seen at 17 μM SU5402 were apparent. The embryos were then incubated for a further 24 h to stage 24 and analysed for changes to the morphology of the second arch. In the DMSO injected embryos (CONT), the second arch has established its characteristic caudal projection (elongated second arch with characteristic morphology *n* = 18/18) (Figure 3a). However, in the triple blocked embryos (EXP), this was not the case and the posterior second arch had adopted a more rounded morphology and had not established a caudal projection with a leading edge (stunted rounded second arch, *n* = 17/20) (Figure 3b). We also analysed the expression of *CCND2* in the treated embryos (EXP) and there is a loss of expression of this gene in the mesenchyme of these embryos when compared with the controls (CONT) (Figure 3c,d). This suggests that *BMP7*, *FGF8* and *SHH* are driving the expansion of the second arch and do so through the stimulation of proliferation in the mesenchyme.

### 3.3 The internalisation of the third and fourth pouches is an independent process

**FIGURE 4:**
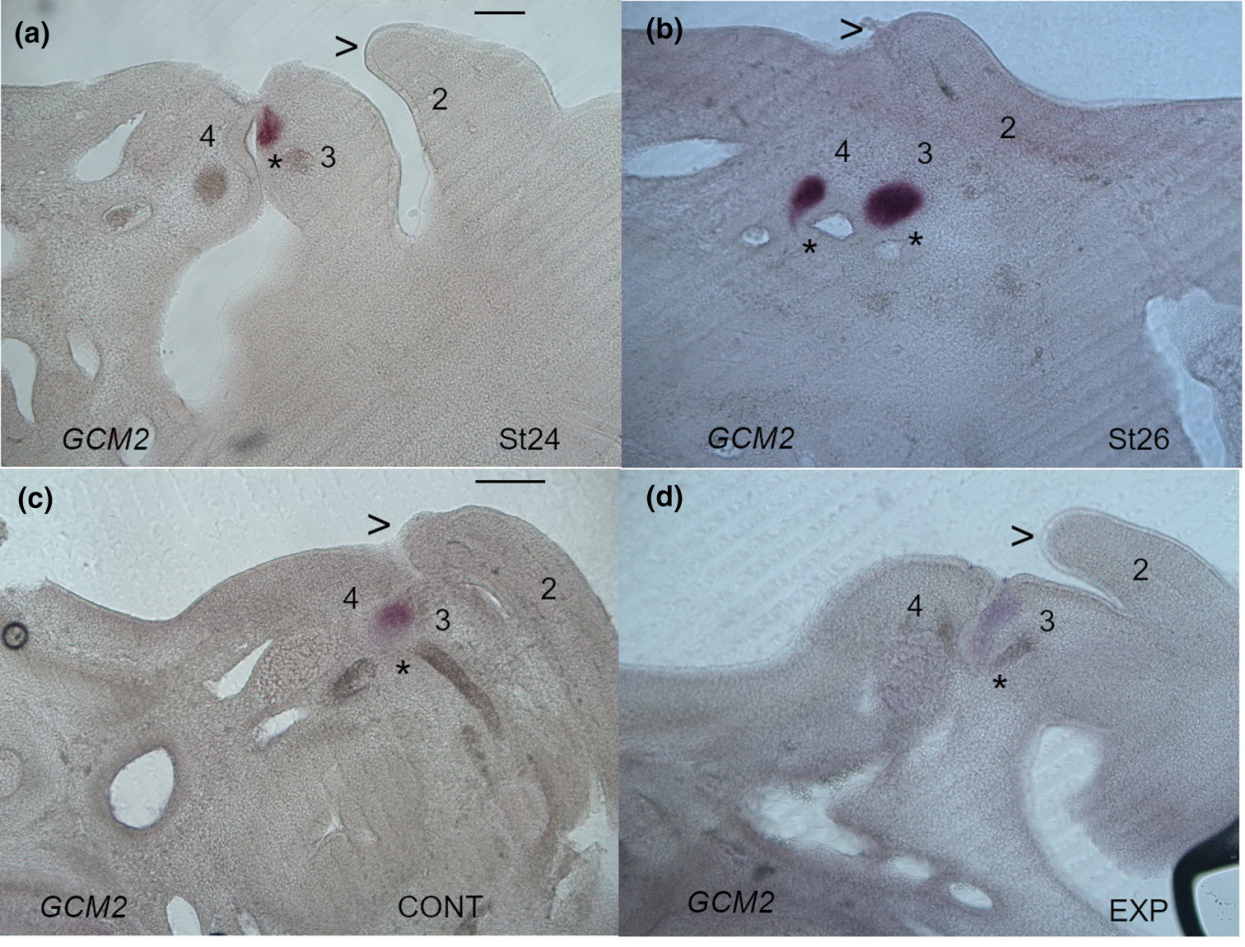
The internalisation of the third and fourth pouches does not depend on the expansion of the second arch. The internalisation of the third and fourth pouches occurs between stages 24 and 26. At stage 24 the third pouch, highlighted via expression of *GCM2*, can be seen to be in contact with the overlying pharyngeal cleft (a). However, by stage 26 the third and fourth pouches, highlighted via *GCM2* expression, have moved internally (b). It can also be seen that between stages 24 (a) and 26 (b) the second arch (2) has expanded over the posterior pharynx. The leading edge of the second arch is highlighted (<). In embryos, that have been injected with DMSO (c) the second arch has projected caudally and can be seen to be covering the earlier site of the fourth pouch which has now internalised. However, in embryos that have been injected with the triple block, the second arch has not extended as far, stopping short of the position of the fourth pouch but this structure has still internalised (d). The “*” highlights the sites of *GCM2* expression. In all panels, anterior is to the right. The pharyngeal arches are numbered, 1, 2 and 3. Scale bar—100 μm.

A key event in the remodelling of posterior pharyngeal arches is the internalisation of pouches three and four, and thus the loss of the posterior arches as individual entities. This process is believed to be driven by the expansion of the second arch and its fusion with the subjacent tissue. We documented the internalisation of the third and fourth pouches through an analysis of *GCM2* expression. GCM2 is a transcription factor that is required for the development of the parathyroid gland, which has its origins in pouches three and four, and thus it is a robust marker of these structures (Okabe & Graham, 2004). At stage 24, the third and fourth pouches are still abutting the overlying pharyngeal clefts and the second arch has not projected over these regions (Figure 4a). However, by stage 26, both the third and fourth pouches are to be found internally and the second arch is overlying the posterior pharynx (Figure 4b).

We have shown that blocking signalling mediated by BMP, FGF, SHH reduces expansion of the second arch. This therefore affords us the opportunity to ask if the internalisation of the third and fourth pouches is dependent on that expansion. To assess this, we injected embryos with the triple block cocktail at stage 22 and then analysed *GCM2* expression 2 days later, at stage 26. We found in the control embryos that the leading edge of the second arch was overlying the internalised pouches (*n* = 16/16) (Figure 4c). In the treated embryos we found that leading edge of the second arch was stunted but that the pouches had still internalised (*n* = 19/19). In both the control and the experimental embryos, the pouches internalised (Figure 4d). Thus, normal development of the second arch is not needed for the remodelling of the posterior arches. Furthermore, we can also conclude that none of these three signalling pathways are needed for the internalisation of pouches three and four.

**FIGURE 5:**
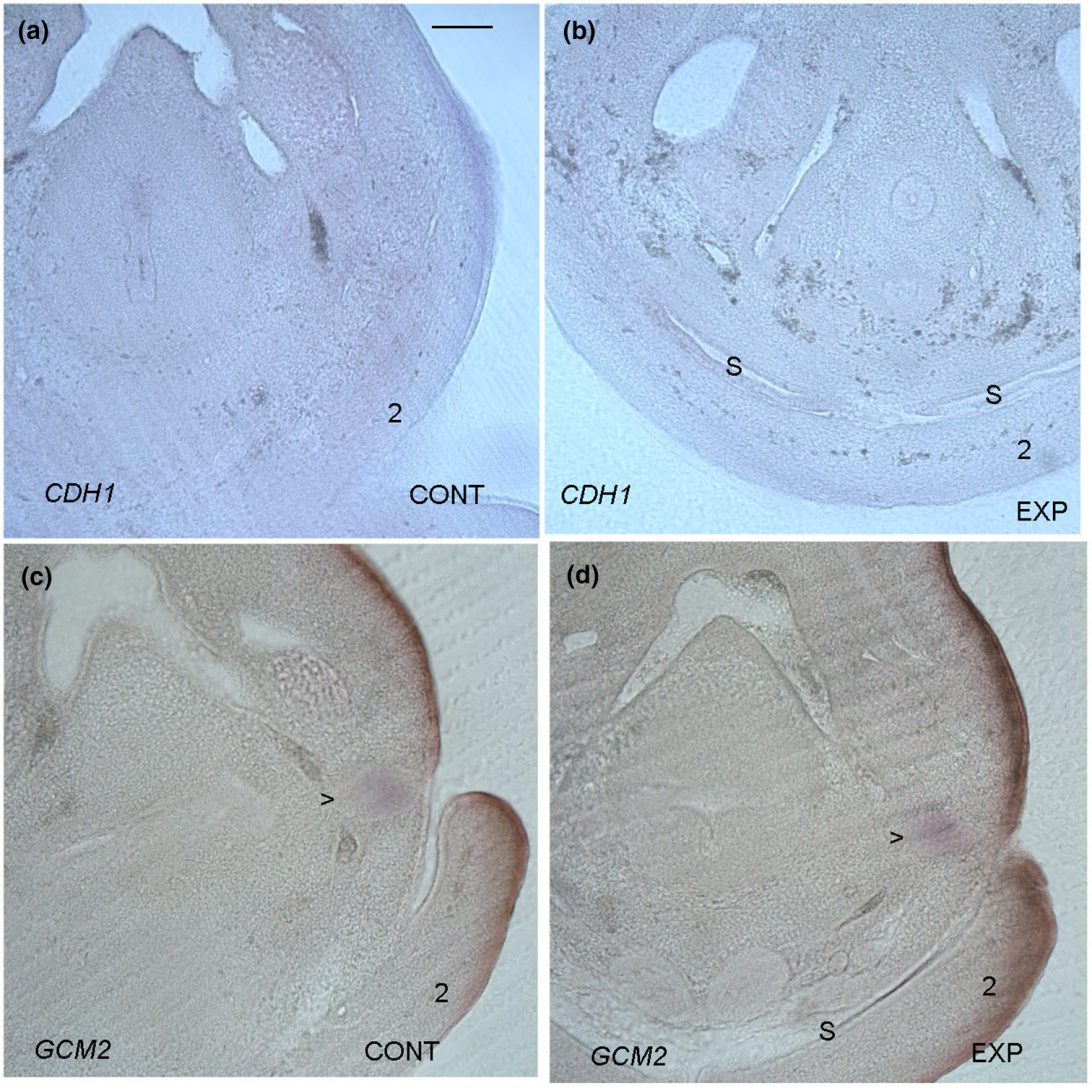
The persistence of the cervical sinus does not interfere with the internalisation of the third and fourth pouches. By stage 28 the cervical sinus which formed earlier between the inner surface of the expanded second arch and the posterior pharynx has been eradicated (a). However, in embryos in which thyroid hormone signalling has been blocked, through sub‐blastodermal injection of amiodarone and methimazole, the sinus persists (S) (b). Causing the sinus to persist has no effect on the internalisation of the third and fourth pouches. In both DMSO treated embryos (c) and in embryos where thyroid hormone signalling has been blocked and the sinus is evident (S) (d) the pouches, as highlighted via *GCM2* expression (>), can be seen to have internalised. All sections are in the transverse plane with dorsal to the top of the image. Scale bar—100 μm.

Another signalling pathway that is known to play a role in pharyngeal remodelling is thyroid hormone. The expansion of the second arch creates a sinus, which is subsequently eradicated, and we have previously shown that thyroid hormone signalling is important here (Richardson et al., 2012). The inhibition of thyroid hormone signalling results in the persistence of the sinus. We therefore assessed if another function of thyroid hormone is to facilitate the internalisation of the third and fourth pouches. To block this pathway, embryos were injected at stage 18 with a cocktail of amiodarone, a thyroid receptor antagonist, and methimazole, which inhibits thyroid hormone synthesis (Richardson et al., 2012), and then analysed for the position of the third and fourth pouches via expression of *GCM2*, at stage 26. We found that while the sinus persists in the treated embryos (persistent sinus *n* = 10/14) versus the controls (no sinus *n* = 10/10) (Figure 5a,b), there was no difference in the positions of the pouches. In both the control and the thyroid hormone signalling blocked embryos the third and fourth pouches had internalised (Figure 5c,d). Thus, the eradication of the sinus has no role in the internalisation and remodelling of the posterior arches and thyroid hormone signalling is also of no importance here.

### 3.4 Termination of expansion of the second arch

**FIGURE 6:**
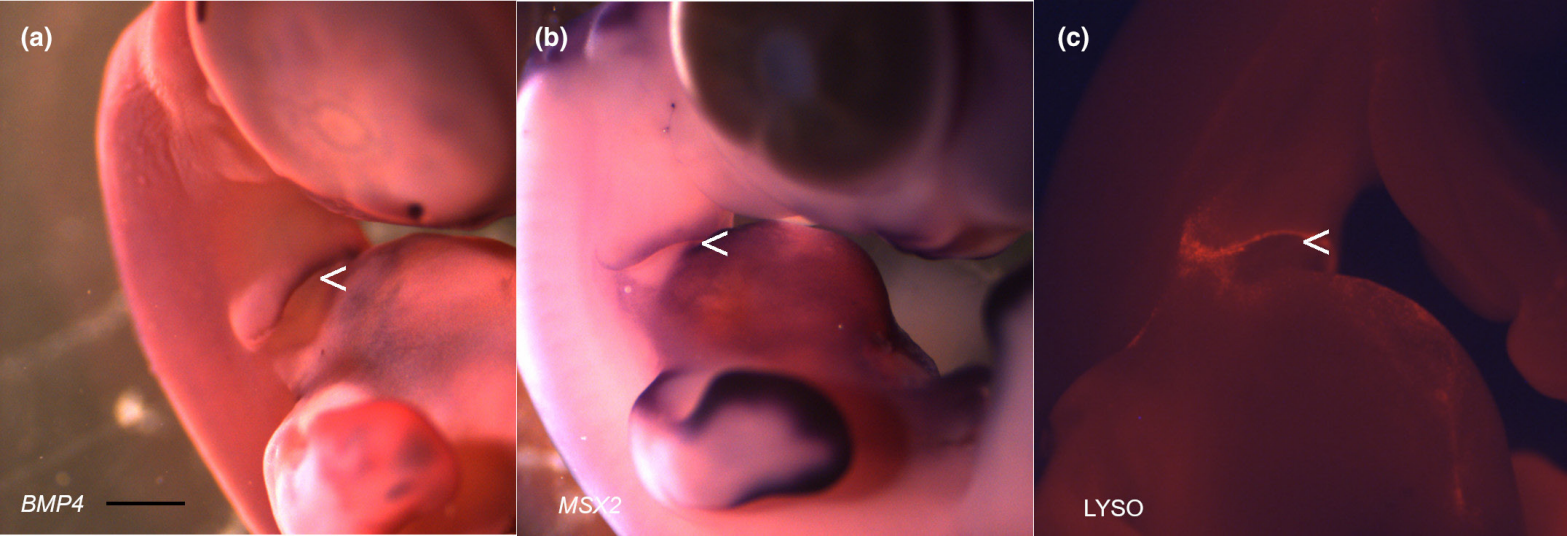
The termination of the expansion of the second arch is associated with a burst of morphogenetic cell death. Large‐scale apoptosis, morphogenetic cell death, is associated with tissue sculpting in several regions of the embryo. Hall marks of this process involve *BMP4* and *MSX2* expression and elevated cell death. The lip of the second arch (<) expresses *BMP4* (a) *MSX2* (b) and is highlighted via Lysotracker staining (c) which picks out apoptotic cells at stage 28. Scale bar—500 μm.

The leading edge of the second arch forms a recognisable morphological entity during the period of extension. However, this protrusion is lost at stage 28 when the expansion is complete. One mechanism underlying such a loss would be cell death and for such a prominent structure—large‐scale morphogenetic cell death. This process has been characterised in other developing systems and it is known to be associated with the expression of *BMP4* and *MSX2* and widespread apoptosis (Graham et al., 1994; Marazzi et al., 1997). We find that this is also true in the pharynx and at a precise point in development, stage 28. Prior to this stage these markers are not expressed at the leading edge. However, at stage 28 the expression of *BMP4* (Figure 6a) and *MSX2* (Figure 6b) are associated with this structure. Lysotracker staining also shows that the leading edge of the second arch is marked by dying cells across its extent (Figure 6c). By stage 29, the expression of *BMP7*, *FGF8* and *SHH* has been lost from the ventral surface of the neck (Figure S2). We conclude that the removal of the lip of the second arch by morphogenetic cell death results in the loss of the cells expressing *BMP7*, *FGF8* and *SHH*, which in turn will result in termination of the growth of the second arch.

## 4 DISCUSSION

The key finding that we present here is that while the remodelling of the pharyngeal arches has always been seen as a singular process driven by the morphogenesis of the second pharyngeal arch with events such as the internalisation of the posterior pouches being subservient to that, this is not the case. Certainly, the expansion of the second arch is the most dramatic event that occurs in the pharyngeal region during the time frame of pharyngeal remodelling, and we show that this is driven by BMP, FGF and SHH signalling. We find that there is localised proliferation in the mesenchyme of the second arch that underlies the posterior epithelial margin, which we show expresses *BMP7*, *FGF8* and *SHH* at high levels during the period of expansion. We have developed a novel approach to simultaneously inhibiting these signalling pathways and we find that when we apply a triple block of these pathways that the caudal expansion of the second arch is curtailed, and there is a loss of proliferation in the underlying mesenchyme. We have then been able to use this perturbation to ask if the internalisation of the posterior pouches is dependent on the expansion of the second arch. We find that it is not. In triple blocked embryos, the internalisation of the posterior pharyngeal pouches occurs as normal even though the morphogenesis of the second arch is abnormal. We can further conclude that the internalisation of posterior pouches is not dependent upon BMP, FGF or SHH signalling. We further show that while thyroid hormone signalling is important for the collapse of the sinus that forms as result of the expansion of the second arch over the posterior pharynx, if this is inhibited and the sinus is not eradicated then this has no consequence for the internalisation of the posterior pouches. Thus, the collapse of the sinus and the fusion of the inner surface of the second arch to the subjacent tissue plays no role in the internalisation of the posterior pouches. Finally, we shown that the termination of the expansion of the second arch is associated with a burst of morphogenetic cell death in the leading edge, which will result in the loss of this tissue and thus the cells expressing the proliferative drivers of expansion.

The expansion of the second arch commences at stage 20 in the chick and continues until stage 28. It thus occurs over a protracted period. We show that this process is associated with localised elevated proliferation within the mesenchymal cells at the posterior of the second arch underlying the posterior epithelial margin. *BMP7*, *FGF8* and *SHH* have been known to be expressed at the posterior margin of the second arch at early stages, and we show that the expression of these genes is maintained at high levels at later stages throughout the period of expansion. We further show that the activities of these pathways are necessary for the localised high levels of proliferation in the underlying mesenchyme and for the expansion. To demonstrate this, we have developed a novel protocol that allows us to introduce inhibitors of these pathways at defined concentrations and at defined times of development. Importantly, this allows us to simultaneously block these three pathways at the critical early point in the expansion phase. When this is done, we find that the expansion of the second arch is reduced, and its morphology altered.

Inputs from multiple signalling pathways are a feature of the development of many embryonic territories and, for example, in both the limb buds and the external genitalia BMP, FGF and SHH signalling are also deployed (Yamada et al., 2006). But what is different with the morphogenesis of the second arch is that the ligands for these pathways are all expressed in the same domain rather than in adjacent sites. Future work will focus on the resolution of the nature of the interactions between these pathways and their precise roles.

The internalisation of pouches three and four occurs between stage 24 and 26 and this is concomitant with the expansion of the second arch over the posterior pharynx. Indeed, these two processes have thought to have been linked. It has been believed that it is the fusion of the expanded second arch with the subjacent tissue of the posterior arches, and the loss of the sinus that forms between these, that resulted in the internalisation of these arches. We were able to test, however, whether that is the case. We find that in triple blocked embryos for BMP, FGF and SHH signalling, that although the expansion of the second arch is limited, pouches three and four still internalise. We also show that when we manipulate thyroid hormone signalling to allow the sinus to persist, and not collapse, that the pouches also internalise. Thus, the normal morphogenesis of the second arch is not required for the internalisation/remodelling of the posterior arches nor is the collapse of the sinus. Furthermore, we can conclude that the internalisation of the posterior arches is not dependent on BMP, FGF or SHH signalling nor on thyroid hormone signalling.

The final step in the morphogenesis of the second arch is the eradication of the termination of expansion and the eradication of the leading edge of this structure. We have observed that there is a correlation between this process and a burst of morphogenetic cell death. Morphogenetic cell death is associated with several important events in the embryo, including the depletion of neural crest cells from rhombomeres 3 and 5 (Graham et al., 1993) and in the interdigital mesenchyme of the limb buds, which then results in the clear delineation of the digits (Yokouchi et al., 1996). In both situations the foci of cell death are associated with the expression of *BMP4* and *MSX2*. Similarly, we find that at stage 28, the leading edge of the second arch is marked by *BMP4* and *MSX2* expression and is associated with high levels of apoptosis. This is likely to have two consequences. One will be to physically remove the leading edge and thus generate the smooth outline of the neck. The second is that it will remove the cells expressing *BMP7*, *FGF8* and *SHH* (Figure S2) the signals that drive the expansion, and thus this process will be terminated. This latter point is a difficult one to establish but it is possible that nature has done the experiment for us. Frill necked lizards, as the name implies, have an erectile frill around their necks and this is generated by the continuous growth of the second pharyngeal arch during late stages of development (Montandon et al., 2019). It has been suggested that the continual growth of the second arch and the generation of the frill is due to the incomplete fusion of the second arch to the underlying posterior pharyngeal arches. While this is possibly the case, we would further suggest that the generation of the frill may also involve the suppression of the morphogenetic death programme that we find associated with the termination of the expansion of the second arch. We would predict that this would result in continued expression of *BMP7*, *FGF8* and *SHH* and thus continued expansion and the formation of the frill. Such a situation would clearly be analogous to the events that are thought to underly the formation of bat wings and the webbing of ducks' feet (Weatherbee et al., 2006; Zou & Niswander, 1996). In both cases, it has been shown that the retention of tissue that would otherwise have been lost through morphogenetic cell death is the result of the de novo deployment of an antagonist of BMP signalling in the regions of morphogenetic cell death, and thus a failure in that programme. We would therefore suggest that this may also be true in frilled necked lizards and allied species.

## AUTHOR CONTRIBUTIONS

AG conceived the project. SP, JR and AG carried out the experimental work and wrote the manuscript.

## Supporting information


Figure S1.

Figure S2.

## Data Availability

The data that support the findings of this study are available from the corresponding author upon request.
