# Supplementary figures and images for: Key separable events in the remodelling of the pharyngeal arches
Source: J Anat. 2023 Feb 23;243(1):100–9. doi: 10.1111/joa.13850 (PMC10273329; doi:10.1111/joa.13850)

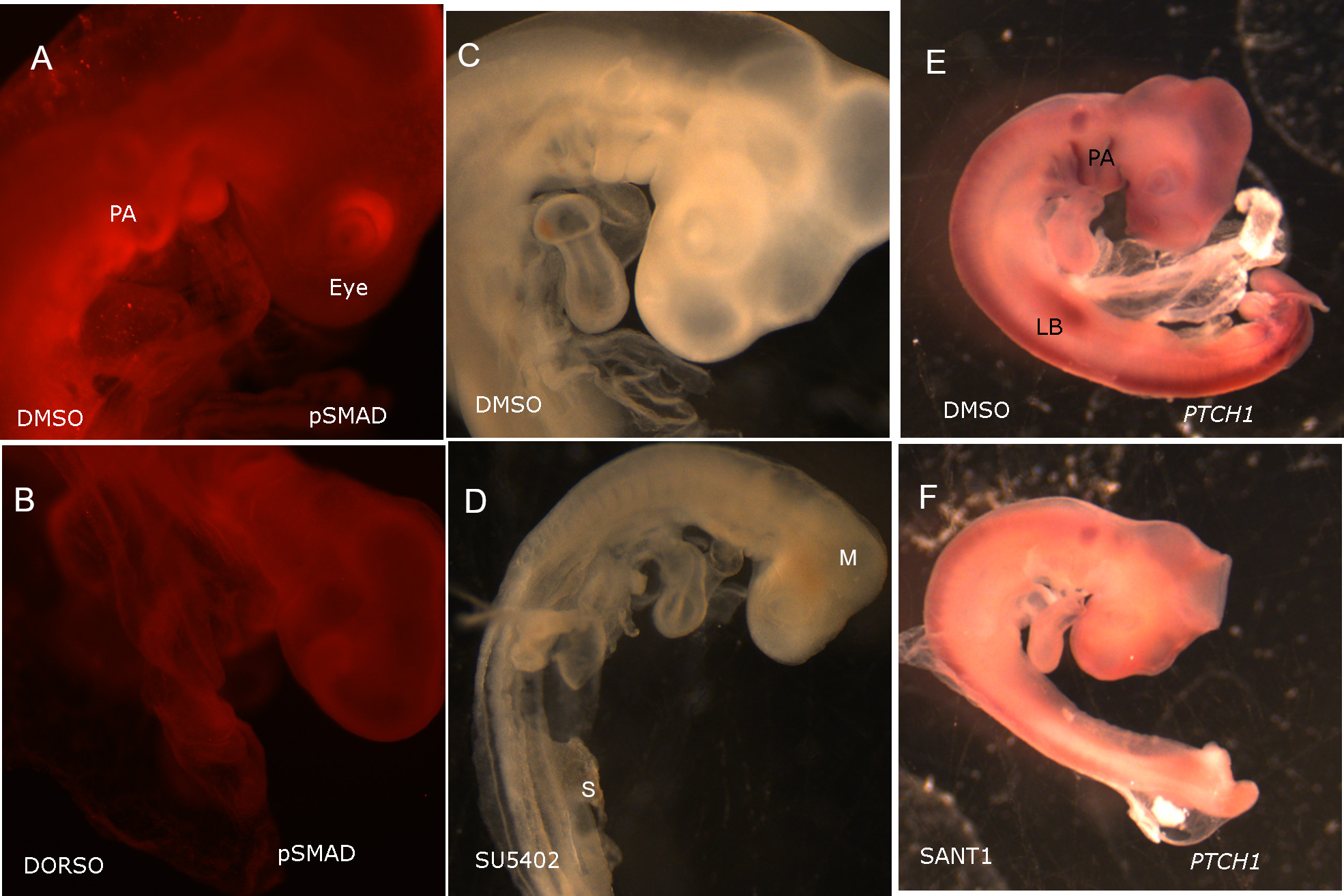

Supplement: Supplementary file 1 — Figure S1. [file JOA-243-100-s002.jpg]

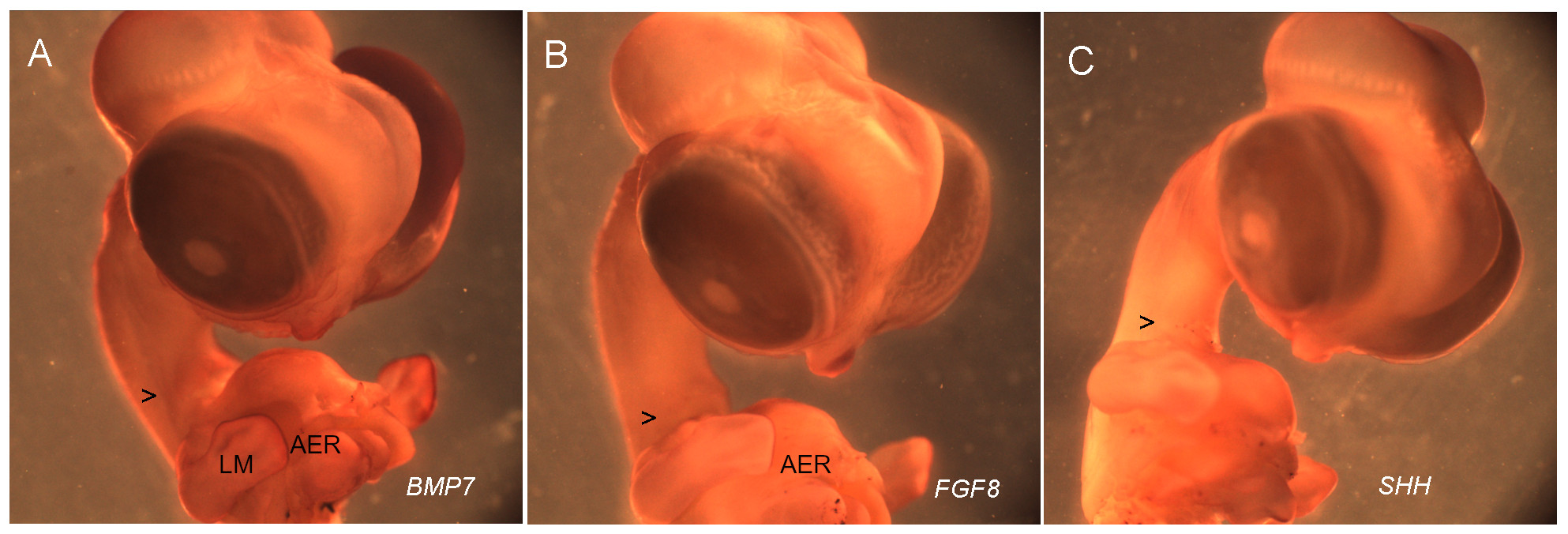

Supplement: Supplementary file 2 — Figure S2. [file JOA-243-100-s001.jpg]
